# A biosemiotic interpretation of certain genital morphological structures in the spiders *Dysdera erythrina* and *Dysdera crocata* (Araneae: Dysderidae)

**DOI:** 10.1007/s12064-023-00404-1

**Published:** 2023-09-13

**Authors:** Joachim Schult, Onno Preik, Stefan Kirschner, Frank Friedrich

**Affiliations:** 1 Department of Biology, History of Science Research Unit, University of Hamburg, Bundesstr. 55, 20146 Hamburg, Germany; 2 Department of Biology, Behavioural Biology Research Unit, University of Hamburg, Martin-Luther-King Platz 3, 20146 Hamburg, Germany; 3 Department of Biology, Teaching and Science Service, Electron Microscopy, University of Hamburg, Martin-Luther-King Platz 3, 20146 Hamburg, Germany

**Keywords:** *Dysdera erythrina*, *Dysdera crocata*, Spiders, Biosemiotics

## Abstract

A biosemiotic approach to the interpretation of morphological data is apt to highlight morphological traits that have hitherto gone unnoticed for their crucial roles in intraspecific sign interpretation and communication processes. Examples of such traits include specific genital structures found in the haplogyne spiders *Dysdera erythrina* (Walckenaer 1802) and *Dysdera crocata* (Koch 1838). In both *D. erythrina* and *D. crocata*, the distal sclerite of the male bulb and the anterior diverticulum of the female endogyne exhibit a striking, previously unreported correspondence in size and shape, allowing for a precise match between these structures during copulation. In *D. erythrina*, the sclerite at the tip of the bulb and the anterior diverticulum are semi-circular in shape, whereas in *D. crocata* they are rectangular. From the perspective of biosemiotics, which studies the production and interpretation of signs and codes in living systems, these structures are considered the morphological zones of an intraspecific sign interpretation process. This process constitutes one of the necessary prerequisites for sperm transfer and the achievement of fertilization. Therefore, these morphological elements deserve particular attention as they hold higher taxonomic value compared to morphological traits of the bulb for which a relevant role in mating and fertilization has not been proven. Thus, an approach to species delimitation based on biosemiotics, with its specific evaluation of morphological structures, provides new insights for the multidisciplinary endeavour of modern integrative taxonomy.

## Introduction

Biosemiotics studies the production and interpretation of signs and codes in living systems (Favareau 2010; Kull 2016). “A semiotic approach in biology means the study of the organisms’ own approach” (Kull 2016, 61). “Own approach” refers to the organisms’ capacity for recognition and differentiation, their intentions, and their knowledge. Mating relies on the recognition of compatible partners. As Kull (2016) puts it, an individual’s “recognition window” is the factor that distinguishes potential partners as either compatible or incompatible. The modern biosemiotic species concept, largely based on Paterson’s recognition concept of species (Paterson 1985, 1988; Masters et al. 1987; Lambert & Spencer 1995; Stamos 2003, 197–199; Mallet 2013, 682–683), lays the focus on those characters, often morphological ones, which serve as signals or signs essential for recognising an appropriate mating partner and achieving fertilisation.

A promising field of applying biosemiotic principles is the genital coupling in spiders with its very complex interactions between the male and female genital structures (Grasshoff 1968, 1973a, 1973b; Huber 1993, 1994a, b, 1998; Uhl et al. 1995, 2007). As Schult et al. (2021) argue, biosemiotically speaking, the fitting between coadapted male and female genital structures can be considered to be a sign whose interpretation leads to or consists of certain effects, such as sperm transfer. Thus, it is not the mechanical fit alone which is important but also that the mechanical fit is a sign in the framework of an indispensable communication process. A description of the mechanical process alone would not suffice to explain its context-dependent meaning. As emphasized by Laubichler (1999), it is crucial to examine the function of a structure within its appropriate context. Therefore, in the specific case of spider reproductive behavior and copulation presented here, it is not solely about the physical fit between two structures, but also about the information and significance that this carries for the organism, as well as the perceptual processes that are likely to be implicated. The recent groundbreaking discovery of neural tissue in the bulb of various other spider taxa (Lipke et al. 2015; Sentenská et al. 2017; Dederichs et al. 2019) adds further weight to this notion.

**Fig. 1:**
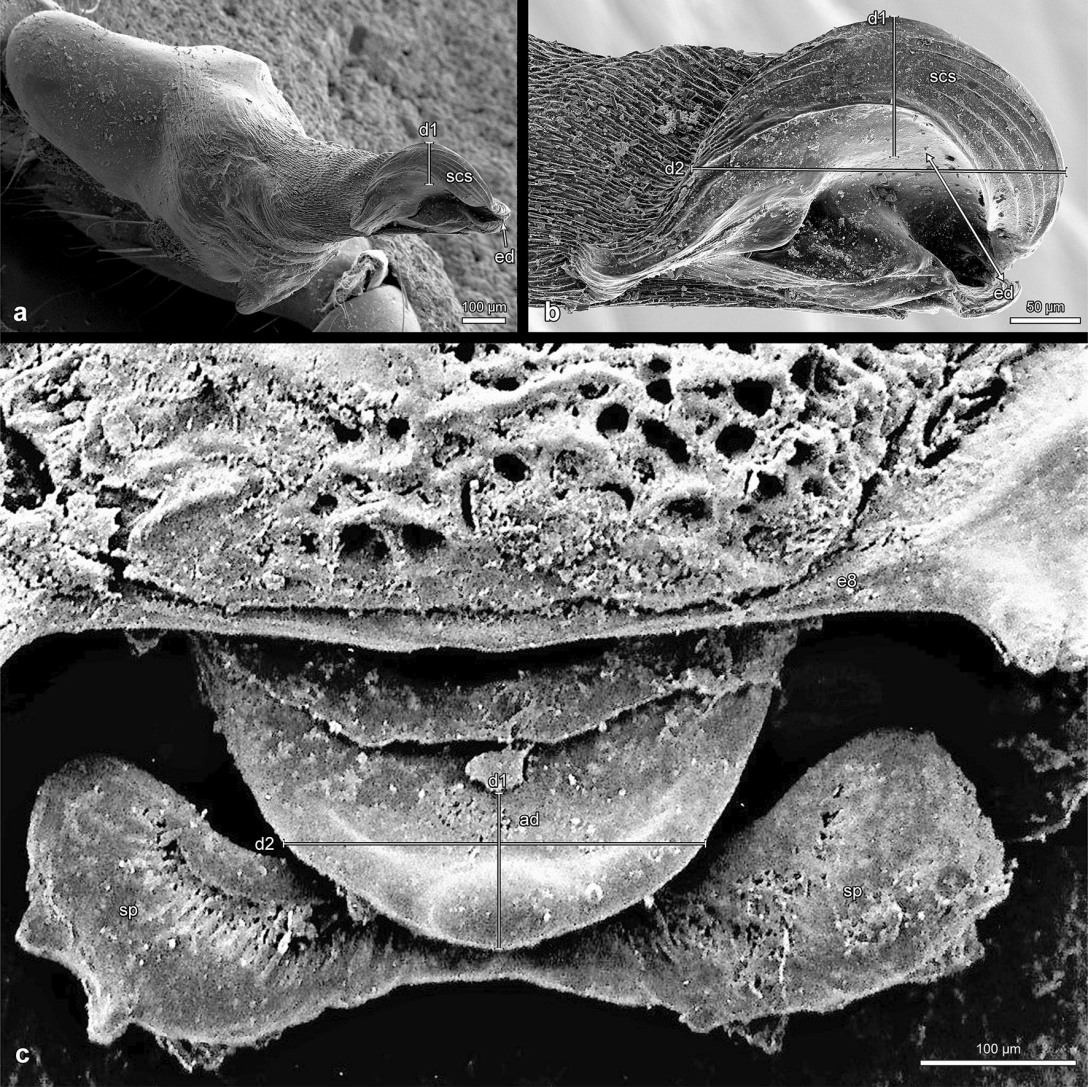
*Dysdera erythrina*, male pedipalp, bulb, and female genitalia, bars d1 (100 μm) and d2 (270 μm) indicating the spatial correspondence between the semi-circular sclerite (*scs*) and the anterior diverticulum (*ad*); **a, b** *scs* = semi-circular sclerite at the tip of the bulb, *ed* = exit opening of the ejaculatory duct; the two pointed arrow in **b** marks the distance (100 μm) between the exit opening of the ejaculatory duct (*ed*) and the edge of the semi-circular sclerite; **c** *ad* = anterior diverticulum of the endogyne, *sp* = spermatheca, *d1* = length of the anterior diverticulum (100 μm), *d2* = width of the anterior diverticulum (270 μm)

**Fig. 2:**
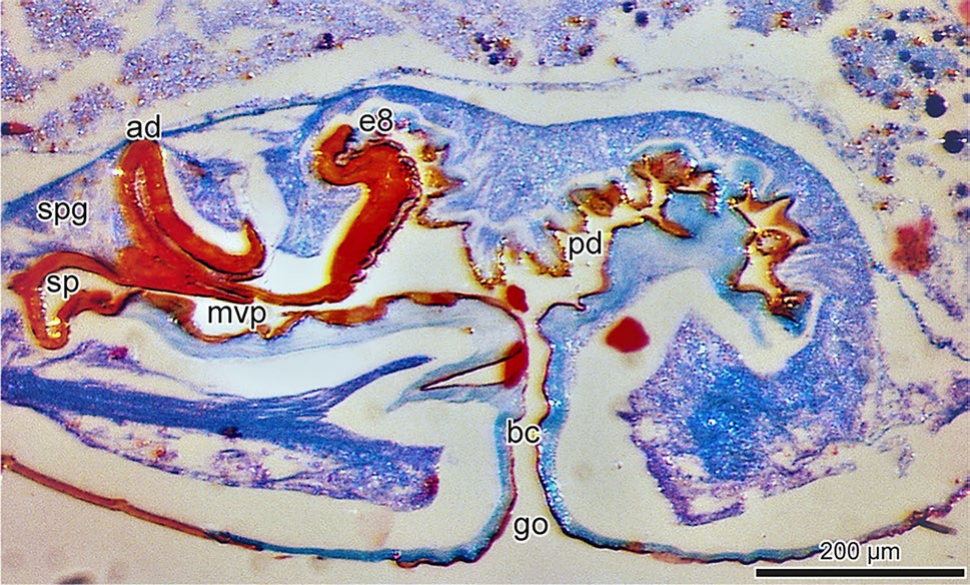
Median longitudinal section of the female genitalia of *Dysdera erythrina*. Abbreviations: *go* = genital opening, *bc* = bursa copulatrix, *pd* = posterior diverticulum, *ad* = anterior diverticulum, *sp* = spermatheca, *spg* = spermathecal gland, *mvp* = median ventral passage, *e8* = entapophysis

Biosemiotics doesn’t stop at asserting functional morphological facts, such as mechanical fits, but goes beyond by asking about their potential meaning in the organism’s “subjective world” (Barbieri 2009). It is about understanding communicative processes between organisms, i.e. the meaning of the signs they use, in the context of their particular perceptive environments or “Umwelten”, a term introduced by Uexküll (1909), which can also be rendered as “self-centred world” or “subjective world” (Kull 2001). In a word, biosemiotics studies what organisms may know and what their types and ways of knowing are (Kull 2012). As Jackson and Cross (2011) conclude in their outstanding and lively description of *Portia fimbriata’s* signals and their reception by potential prey, a human observer at least has to try to interpret spider behaviour from the spider’s point of view. Interestingly, although their approach is a biosemiotic one par excellence, Jackson and Cross don’t use this term anywhere, which indicates that the principles of biosemiotics are still much too little known in their potential fields of application. Concerning mimicry and visual communication, the importance of biosemiotics for morphology has already been exemplified (Kleisner 2008a, 2008b, 2015; Kleisner and Maran 2014; Brejcha and Kleisner 2016; Maran 2017; Kleisner and Saribay 2019). The purpose of our paper is to demonstrate that a biosemiotic approach to interpreting morphological data provides an additional tool to reveal species-specific morphological traits whose key role in intraspecific communication processes and the achievement of fertilisation has been overlooked so far. Certain genital structures of the spider species *Dysdera erythrina* (Walckenaer 1802) and* Dysdera crocata* (Koch 1838) will serve as examples. *D. erythrina* and *D. crocata* belong to two distinct groups of morphologically very similar species (Řezáč et al. 2007/2008, 2018) (Figs. 1 and 2).

**Fig. 3:**
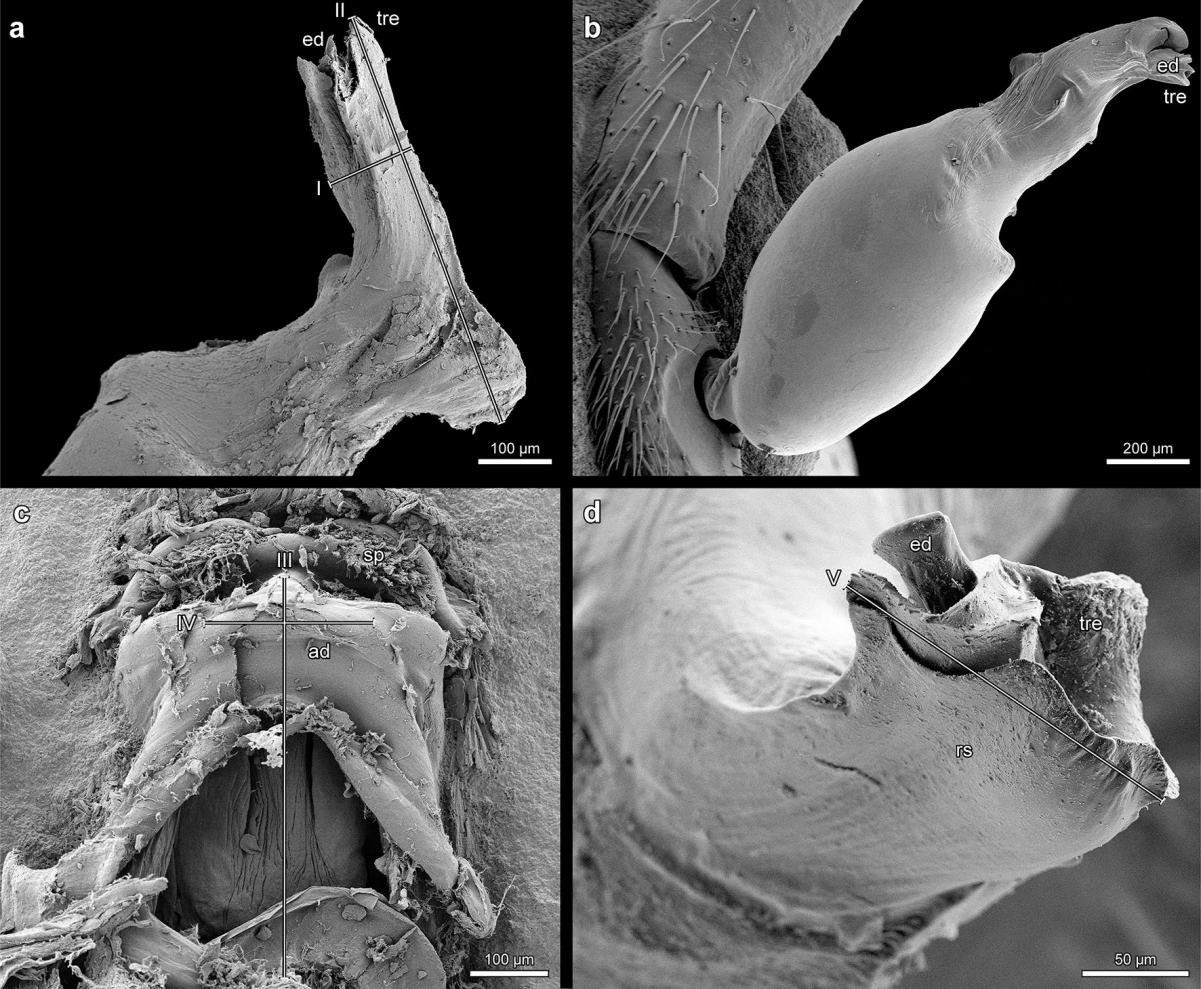
*Dysdera crocata*, bulb (**a**, **d**), male pedipalp (**b**), endogyne (**c**), and spatial correspondence between the rectangular sclerite (r*s*) at the tip of the bulb and the anterior diverticulum (*ad*); **a**, **b** *tre* = distal tricuspid end of the bulb, *ed* = exit opening of the ejaculatory duct, dimensions: I: 123 μm, II (length of the distal portion of the bulb): 588 μm; **c** *ad* = two-tiered anterior diverticulum of the endogyne, *sp* = spermatheca, dimensions: III (length from the tip of the anterior diverticulum to the level of the epigastric furrow or insertion opening): 542 μm, IV: 200 μm; the length of the distal portion of the bulb (**a**, II) corresponds to III; **d** *rs* = rectangular sclerite at the distal tricuspid end (*tre*) of the bulb, *ed* = exit opening of the ejaculatory duct, V measures 188 μm; the width of *rs* (**d**, V) corresponds to the width of the dorsal part of *ad* (**c**, IV)

**Fig. 4:**
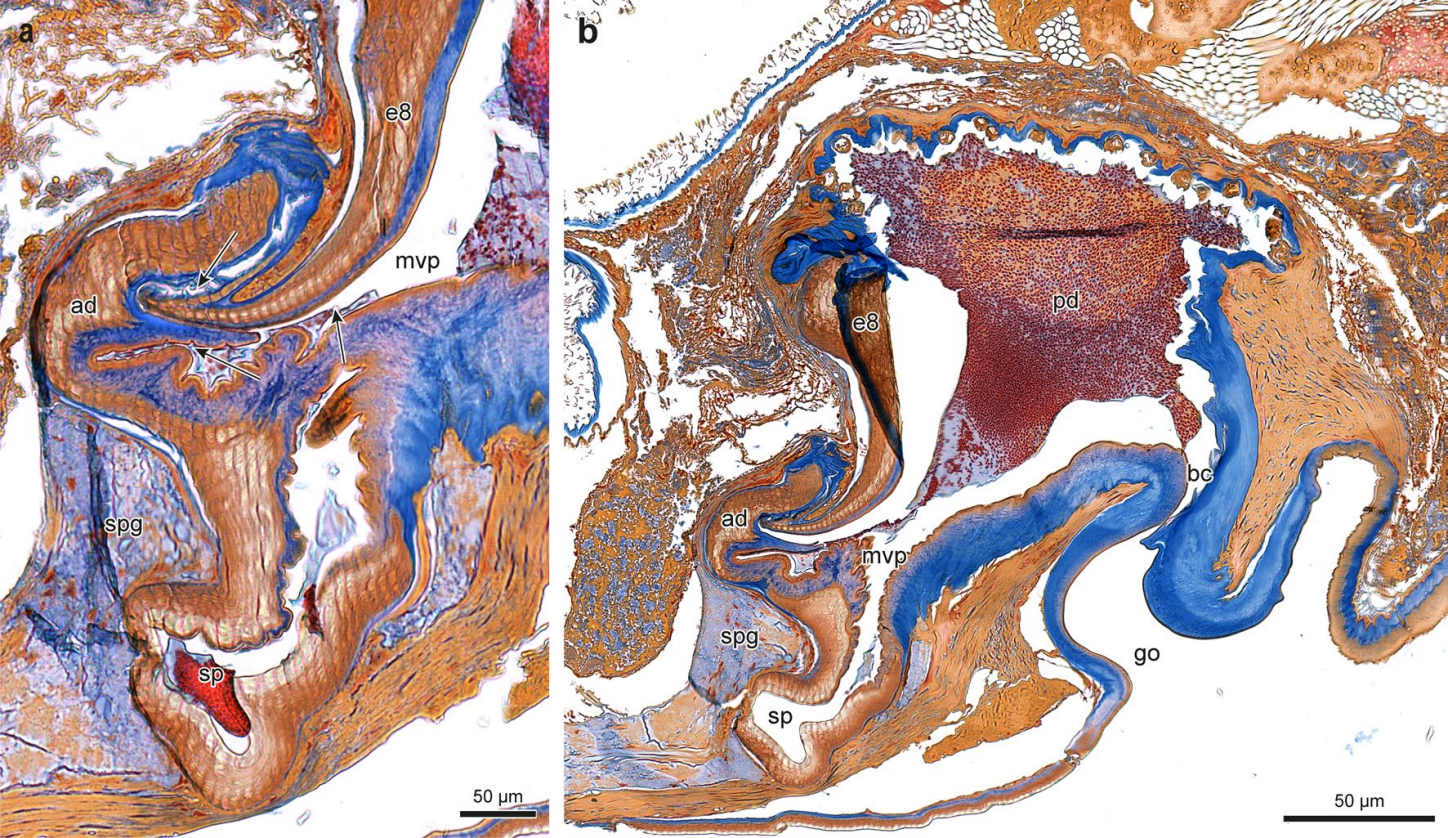
Median longitudinal section of the female genitalia of *Dysdera crocata*, **a** distal area of the endogyne, **b** overview, *go* = genital opening, *bc* = bursa copulatrix, *pd* = posterior diverticulum, *ad* = anterior diverticulum, *sp* = spermatheca, *spg* = spermathecal gland, *mvp* = median ventral passage, *e8* = entapophysis 8. The arrows in **a** indicate the three sites of contact with the tricuspid tip of the bulb

The female and male copulatory organs have always been the focus of spider taxonomy and species characterization. In what follows we use the term “endogyne” instead of “vulva” to denote the female copulatory apparatus in haplogyne spiders, in analogy to “epigyne” in entelegyne spiders (Mkheidze 1972; Arnedo & Ribera 1997; Fomichev & Marusik 2020; Deeleman-Reinhold and Deeleman 1988, 144; Zamani et al 2023). While the characteristic shape of the tip of the male bulb’s embolus in *Dysdera erythrina* has been noticed (Arnedo et al. 2009; Řezáč et al. 2018), its possible function during copulation has been touched upon only marginally (Řezáč et al. 2018, 20). The same applies to publications on *Dysdera crocata* (for example Cassar and Řezáč 2021, 83 Fig. 3a; Cooke 1966, 38 Fig. 4; de Luna et al. 2022, 332 Fig. 1 D; Deeleman-Reinhold and Deeleman 1988, 159 Figs. 23–27; Harvey 2009; Kovblyuk et al. 2008, 289 Figs. 1–4, 8–10; Paquin and Dupérré 2003, 71; Řezáč et al. 2007/2008, 435 Figs. 2–4; Trotta 2005, 149 Fig. 32). There are neither detailed illustrations of the distal parts of the bulb of *D. crocata*, nor is there any discussion of the complementarity between the bulb’s tip and structural elements of the endogyne. By contrast, a biosemiotic approach reveals that the embolus’s tip and its corresponding endogynal structure deserve much more attention concerning their role in fertilisation and their taxonomic value.

## Materials and methods

### Material examined

*Dysdera erythrina* (Walckenaer, 1802): 2 adult females (Fig. 1c), collected by Joachim Schult in Freiburg i. Br. (Germany) in 1997. They were determined by J. Schult and deposited in the Museum of Nature and Environment (Lübeck, Germany). 1 male adult (Fig. 1a–b) from the Centrum für Naturkunde (CeNak, Hamburg, Germany), collected by W. Bösenberg and determined by him in 1900 (list no. 814). 1 adult female (Fig. 2), collected by Stefan Lauterbach in Bestwig (Sauerland, Germany) in 2018 and determined by him. This specimen is deposited in the Institute of Zoology of the University of Hamburg.

*Dysdera crocata* (Koch 1838): 2 adult females, collected by Stefan Lauterbach in Frankfurt (Germany) in 2017 (Fig. 3c) and in Karlsruhe (Germany) in 2006 (Fig. 4a, b), and determined by him. 2 adult males, collected by Jürgen Gruber in Wien, Liechtensteinpark (Austria) in 1982 (NHWM 17016, Fig. 3a) and in Wien, Augarten (Austria) in 1997 (NHWM 19672, Fig. 3b, d), and determined by him.

### Specimen preparation and imaging

For morphological investigation male pedipalps of *Dysdera erythrina* and *Dysdera crocata* were isolated, embedded in Hoyer’s medium (Kraus 1984b), and investigated light microscopically using a Leitz Stereomicroscope and a Leitz Orthoplan with interference contrast.

Morphological investigation of the longitudinal sections of the female genitalia of *Dysdera erythrina* stained with aniline blue and nuclear fast red staining was carried out with a Leica M2 16/ Camera Leica DFC 320/ Leica Application Suite 4.6.0. The longitudinal sections of the female genitalia of *Dysdera crocata* were prepared and stained with AZAN by the company MORPHISTO Ltd., Offenbach (Germany). For scanning electron microscopy the male parts of *Dysdera erythrina* were fixed in 70% ethanol, dehydrated in serial dilutions of ethanol, transferred in acetone, critical point dried (Leica CPD 300), and coated with carbon (Leica ACE 600). The preparations were embedded in Hoyer’s mixture (Crabill 1958) between two coverslips and investigated in a Zeiss LEO 1525 scanning electron microscope (SEM). Furthermore, a SEM photograph made by J. Schult in 1983 has been reinterpreted based on a biosemiotic approach (Fig. 1c). While this older photo does not meet current standards in every respect, the structures in question are clearly visible. The SEM (Stereoscan S4, Cambridge Scientific Instruments, Ltd.) was a loan from the DFG (Deutsche Forschungsgemeinschaft, German Research Foundation) to the former working group “Hard Body Morphology” at the Institute of Zoology, University of Hamburg.

## Results and discussion

The tip of the bulb of *Dysdera erythrina* is dominated by a semi-circular sclerite (scs) (Fig. 1a–b). This structure, called by Arnedo et al. (2009) and Řezáč et al. (2018) an arch-like ridge or a “large, semicircular expansion”, is considered one of the main synapomorphies of the *Dysdera erythrina* species complex (Řezáč et al. 2018, 20). In our view its semi-circular shape should be referred to in its denomination. Therefore, we prefer in what follows to call this structure a semi-circular sclerite. In Fig. 1b the exit opening of the ejaculatory duct (ed) is recognisable at a distance of 100 μm from the edge of the semi-circular sclerite.

The dorsal view of the female genitalia (Fig. 1c) reveals the bilobed spermatheca (sp) and the anterior diverticulum (ad). Figure 2 gives information on the course of the genital cavity and the relative position of the spermatheca, the anterior diverticulum (ad), and the posterior diverticulum (pd).

*Dysdera erythrina* (Fig. 2) possesses a single median genital opening (go), a narrow slit leading anteriorly from the epigastric furrow, and opening into the bursa copulatrix (bc). The bursa is lined with a cuticular layer. Ventrally it is much thickened and in parts sclerotised. Posteriorly the bursa dilates into a flexible walled diverticulum (pd). The posterior diverticulum is lined with a thin cuticular layer pierced by chitinous tubercles. Anterodorsally the posterior diverticulum’s wall fuses with the entapophysis (e8) (genital 8th somite) (Figs. 1c, 2), a transverse sclerotised bar that forms an important structural support for the endogyne. In addition, the entapophysis forms the dorsal wall of the bursa copulatrix (bc). Anteriorly the bursa ends blindly in a sclerotised diverticulum (ad), which in *Dysdera erythrina* is clearly pronounced (Figs. 1c, 2). An anterior median ventral passage (mvp) leads from the bursa into the heavily sclerotised bilobed spermatheca.

It is of particular interest and has been unnoticed so far that the anterior diverticulum corresponds in shape and size to the semi-circular sclerite located at the bulb’s tip (see the bars d1 and d2 in Fig. 1a–c). Furthermore, the distance between the exit opening of the ejaculatory duct (ed) and the edge of the semi-circular sclerite (Fig. 1c, two pointed arrow) coincides with the distance between the distal end of the anterior diverticulum and the beginning of the ventral-median passage leading to the spermatheca. Hence, from the morphological conditions shown in Figs. 1 and 2 it can be inferred that the bulb’s distal sclerite is firmly anchored in the anterior diverticulum during copulation. Introducing the bulb’s semi-circular sclerite into the anterior diverticulum makes the exit opening of the ejaculatory duct reach the beginning of the duct leading to the spermatheca. This arrangement provides optimal conditions for sperm transmission.

The distal structure of the bulb of *Dysdera crocata* is completely different from that of *Dysdera erythrina*. As we have observed, in *D. erythrina*, the sclerite located at the bulb’s tip is semi-circular (Fig. 1b), whereas in *Dysdera crocata*, it is rectangular with a distal tricuspid end (*tre*) (Fig. 3a, d). The shape of the anterior diverticulum in *Dysdera crocata* is also rectangular (Fig. 3c), corresponding in size and shape to the distal portion of the bulb. The length of the distal portion of the bulb (Fig. 3a, II) corresponds to the length from the tip of the anterior diverticulum to the level of the epigastric furrow or insertion opening (Fig. 3c, III), while the width of the sclerite (Fig. 3d, V) matches the width of the dorsal part of the anterior diverticulum (Fig. 3c, IV).

The dorsal view of the female genitalia of *D. crocata* (Fig. 3c) reveals the bilobed spermatheca (*sp*) and the anterior diverticulum (*ad*). In contrast to *Dysdera erythrina*, the anterior diverticulum of *Dysdera crocata* has a two-tiered structure (Fig. 3c). The course of the genital cavity and the locations of the spermatheca (sp), anterior diverticulum (*ad*), and posterior diverticulum (*pd*) are shown in Fig. 4. The solitary median genital opening (*go*) in *Dysdera crocata* (Fig. 4) is a small slit that extends anteriorly from the epigastric furrow and opens into the bursa copulatrix (*bc*).

**Fig. 5:**
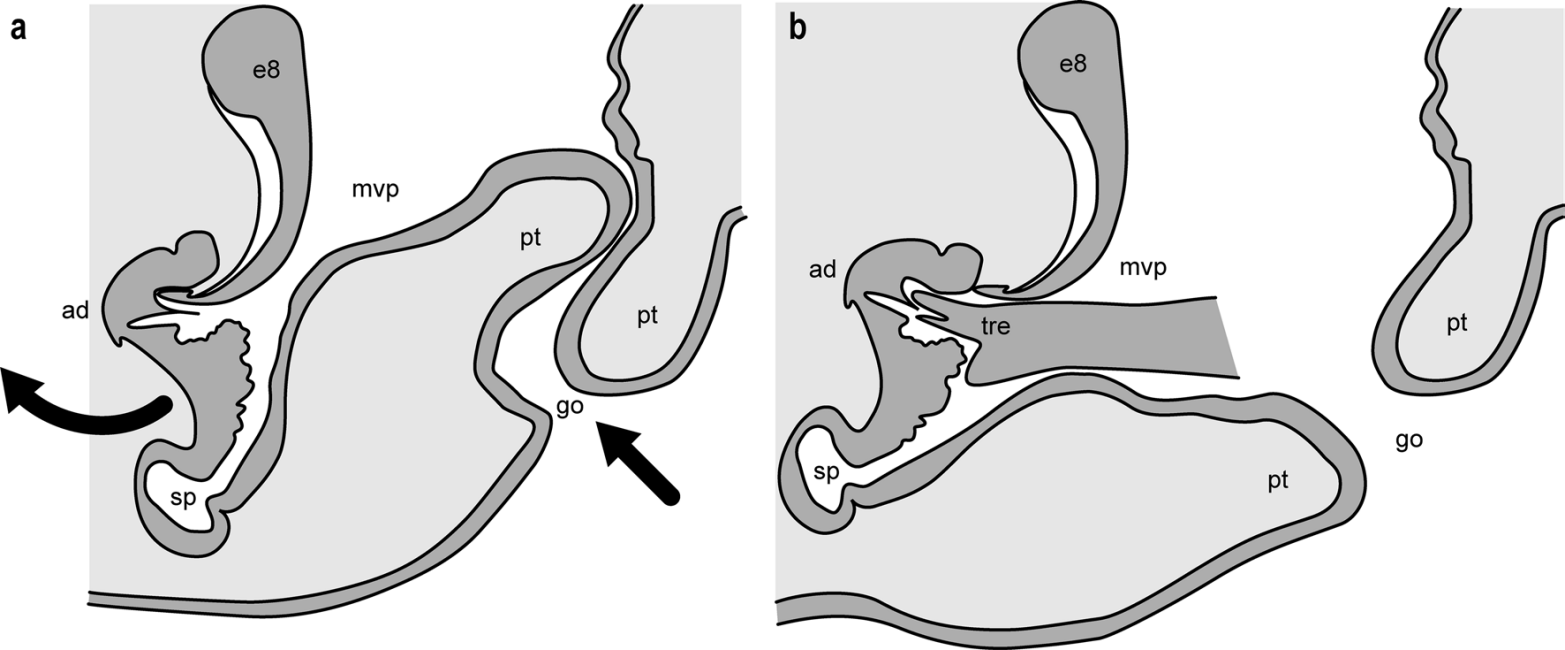
Hypothetical sketch depicting the change in shape of the endogyne of *Dysdera crocata* when the distal part of the bulb is inserted. During this process, the bursa copulatrix dilates and the apical wall of the endogyne is pushed forward. Curved arrow: direction of movement of the front wall; straight arrow: insertion of the bulb. Only the distal tricuspid end of the bulb was considered. Abbreviations: *go* = genital opening, *ad* = anterior diverticulum, *sp* = spermatheca, *mvp* = median ventral passage, *e8* = entapophysis 8, *tre* = distal tricuspid end of the bulb, *pt* = protuberances of the anterior and posterior walls of the insertion duct

Unlike *D. erythrina*, the bursa copulatrix in *D. crocata* is not directly passable. Two opposite protuberances (Fig. 4b), each on the anterior and posterior walls, overlap and thus close this entrance canal. Only the insertion of the distal part of the bulb into the canal opens it by folding the anterior wall forward (Fig. 5a, b). The arrows in Fig. 4a indicate the three sites of contact with the tricuspid tip of the bulb. An anterior median ventral passage (*mvp*) leads from the bursa into the heavily sclerotised spermatheca (Fig. 4).

Male spiders lack primary copulatory organs. Instead, their pedipalps are modified to facilitate sperm transfer. The pedipalps of males are altered in the distal section to serve as a secondary copulatory organ. This secondary copulatory organ varies in complexity among different subgroups of the Araneae (Comstock 1910; Schult 1983; Eberhard & Huber 2010). In haplogynous spiders, males possess relatively simple structured pedipalps, and correspondingly, females have comparatively uncomplicated copulatory organs.

Structural distinctions in the pedipalps and endogyne have consistently been employed to differentiate species. These specialized structures may help prevent interbreeding between different species, bearing in mind that hybridization may also be hindered at the behavioral level. Therefore, any novel findings concerning the morphology and function of spiders’ sexual organs hold significant taxonomic relevance.

This paper demonstrates a striking, previously undocumented correspondence between the size and shape of the bulb’s distal sclerite and the anterior diverticulum in both *D. erythrina* and *D. crocata*. The specific morphology of the distal sclerite of the bulb in *Dysdera erythrina* and *Dysdera crocata* implies that when the sclerite accurately fits into the anterior diverticulum, the exit opening of the ejaculatory duct (Figs. 1a, b; 3a, b, d) reaches the beginning of the duct leading to the spermatheca. By contrast, Řezáč et al. (2018, 20) state, assuming that the arch-like ridge in *D. erythrina* obtains quite a different positioning during copulation: “The opening of the sperm duct falls close to the opening of the channel leading to the spermatheca, whereas the frontal lobe of the arch-like ridge of the bulb pokes at the ventral wall.” However, if this were the case, the exit opening of the embolus could not reach the beginning of the duct leading to the spermatheca. Moreover, the exact spatial correspondence between the semi-circular shape of the arch-like ridge and the shape of the anterior diverticulum would be a mere coincidence.

Semiotically speaking, this correspondence between the bulb’s semi-circular sclerite and the anterior diverticulum is a sign whose interpretation by the male leads to the effect of sperm transfer. This explanation is fundamentally rooted in Peirce's concept of a triadic relation, encompassing the subsequent elements: 1. the object as the entity signified, 2. the sign or representamen as the element signifying, and 3. the interpretant, which denotes the impact generated by the sign on an interpreter of it (Short 2004). In this context, the correspondence between the bulb’s semi-circular sclerite and the anterior diverticulum serves as the representamen or sign-vehicle, the interpretation of which results in sperm transfer, representing the interpretant. The extent to which the female also engages in this interpretive process remains uncertain.

While some biosemioticians have already employed Peirce’s terminology at the molecular or cellular level, such as in describing the sequence of catalyst binding on the template as an immediate interpretant (Deacon 2021), Barbieri, within his “code model of semiosis” and “Code Biology”, confines the application of the term “interpretation” to scenarios involving an “abduction” executed by neural networks, essentially a “mental leap beyond appearances” (Barbieri 2009, 2015, 2019). “Abduction” is another concept introduced by Peirce, signifying an extrapolation from limited data, introducing a third logical category in addition to the traditional categories of induction and deduction. Regarding the principles of code biology, the alignment between coadapted male and female genital structures could be contemplated as a form of morphological code, as suggested by a reviewer of this article.

From the anchoring of the bulb’s tip inside the anterior diverticulum, it can be inferred that sperm can only be ejaculated into the anterior diverticulum. The assumption of other authors (Cooke 1966; Uhl 2000) that sperm is discharged into the posterior diverticulum without reaching the spermathecal lumen and that very likely sperm has to be moved from the posterior diverticulum to the spermathecae after copulation has taken place, cannot be confirmed by the copulation behaviour or the copulation mechanics. Quite the contrary: the fact observed by Uhl (2000), that the posterior diverticulum acts as a sperm storage organ, has to be explained by an opposite direction of post-copulatory sperm translocation, namely from the spermathecae to the posterior diverticulum. Considering the endogyne as a whole, only some of its morphological elements can be explained by their function in copulation and sperm collection. Above all, the bow-shaped entapophysis VIII does not seem to be associated with these functions. Its proper functional meaning and semiotic status still have to be identified.

In a biosemiotic context, the bulb’s semi-circular (*Dysdera erythrina*) or rectangular (*Dysdera crocata*) sclerite and the anterior diverticulum form an intraspecific communication zone during copulation. We suppose that a mechanical mismatch between these structures would constitute a reproductive barrier during copulation, that is, a barrier that operates after mating has begun, but before gametes make contact, as defined by Wojcieszek and Simmons (2013). It is decisive to realise that such a reproductive barrier doesn’t exist on the mechanical level alone, but also on the communicative level, as the sign normally produced by the mechanical fit between the bulb’s distal sclerite and the anterior diverticulum is now missing. Thus, the intraspecific communication and sign-interpreting processes during copulation as described in *Dysdera erythrina* and *Dysdera crocata* can be regarded as necessary prerequisites – among other processes and mechanisms influencing fertilisation success, such as sperm competition and cryptic female choice (Eberhard 2004; Firman et al. 2017; Herberstein et al. 2011; Schneider & Andrade 2011; Uhl & Vollrath 1998) – for successful copulation and sperm transfer.

The shape of the distal semi-circular sclerite or arch-like ridge of the male bulb in *Dysdera erythrina* is completely different from the form of the male bulb’s distal end in *Dysdera* species not belonging to the *Dysdera erythrina* species complex (Arnedo and Ribera 1997; Arnedo and Ribera 1999; Arnedo et al. 2000, 2007, 2009; Crespo et al. 2021; Deeleman-Reinhold and Deeleman 1988; Le Peru 2011, 223–228; Macías-Hernández 2010; Wunderlich 1991, 530–541; Řezáč et al. 2007/2008; Řezáč et al. 2014; Ribera 2004; Zamani et al. 2023). The same applies to *Dysdera crocata* with its rectangular distal sclerite of the bulb. Within the *Dysdera erythrina* species complex, for which the presence of an arch-like ridge is considered a synapomorphy (Řezáč et al. 2018, 20), Řezáč et al. (2018, 53) distinguish two species-specific morphological characters concerning the arch-like ridges, namely (1) whether the relative size of the membranous patch in the apical part of the arch-like ridge is relatively large or relatively small or inconspicuous and (2) whether the proximal part of the arch-like ridge protrudes from the outline of the bulb in hind view (when a posterior apophysis is inclined to an observer) almost at a right angle or gradually. However, it is astonishing that Řezáč et al. speak of species-specific characters in these cases, although according to their table, none of these characters is restricted to one *Dysdera* species but present in several species. Furthermore, since the anterior diverticulum and the semi-circular or rectangular sclerite of the bulb in *Dysdera erythrina* and *D. crocata* have an exact mechanical fit, the variations in size and shape of the distal sclerites of the bulbs in other *Dysdera* species and their potential correspondences to morphological details in the anterior diverticulum deserve more attention. Thus, further comparative studies are needed for a final determination of the taxonomic status of these structures.

The physiological basis of the intraspecific communication and sign-interpreting processes as described above is still unknown. Interestingly, in the entelegyne spider *Philodromus cespitum*, it has recently been shown that the bulb, commonly considered a numb organ (Eberhard and Huber 2010), possesses neural tissue and a multisensillar sensory organ situated close to the base of the embolus (Sentenská et al. 2017). Furthermore, neurons are associated with two glands within the bulbus, the fundus gland and the embolus gland. Sentenská et al. (2017) suggest that “sperm expulsion may be triggered when the sensory organ sends information about the correct positioning of the pedipalp during mating. This afferent transmission may cause the fundus gland to release the substance from its reservoir into the spermophor lumen to flush out the seminal fluid stored therein.“ Pioneer work in this area was done by Lipke et al. (2015) who for the first time documented the presence of neurons and a nerve inside the palpal bulb of a spider, the haplogyne Tasmanian cave spider *Hickmania troglodytes*. As „the arrangement of the nerve and neurons in *H. troglodytes* and *P. cespitum* is nearly identical although the bulbi differ considerably between these two species “, which belong to distantly related taxa, Sentenská et al. (2017) assume “that the presence and arrangement of the neural tissue represents a ground pattern for all Araneomorphae and possibly for all spiders. “Their assumption was impressively confirmed by Dederichs et al. (2019) corroborating that in another nine spider taxa, there is a bulb nerve, which is a distal branch of the palpal nerve. Moreover, Dederichs et al. (2019) found afferent or efferent neurite bundles projecting from the bulb nerve into various parts of the palpal organ, a sensory organ at the base of the embolus in several of the investigated taxa and nervous tissue close to the glandular tissue of the spermophor. Future research has to clarify whether the bulbs of *Dysdera erythrina* and *Dysdera crocata* are also innervated. If so, an explanation would be at hand as to how the mechanical fit between the bulb’s distal sclerite and the anterior diverticulum, which serves as a sign that the embolus and its exit opening of the ejaculatory duct have reached their correct position for sperm transfer, is perceived. However, any involvement of trichobothria, which are recognized for their function as mechanoreceptors and touch receptors (Gillespie 2009, 944), is ruled out due to the absence of tactile hairs of that kind on the surface of the bulb.

As exemplified by *Dysdera crocata* and *Dysdera erythrina*, a biosemiotic shift from the purely morphological to the communication and information aspect by strictly focussing only on those morphological structures that are essential for intraspecific communication is apt to avoid typological interpretations of morphological structures and the problems usually associated with them (Schult 2004; Schult et al. 2021). In this way, biosemiotics contributes to strengthening morphology, which has experienced a significant decline since the beginning of the twentieth century. On the other hand, biosemiotics also benefits from its application as a kind of heuristic tool in morphology, because a broader acceptance of biosemiotic principles in the life sciences can only be expected when it is clearly demonstrated how biosemiotics is capable of obtaining empirical data and insights that lie beyond the scope of biology operating under conventional research approaches.

The male secondary copulatory organ (palpal bulb) and the female reproductive organ have always played a major role in spider taxonomy. Nevertheless, detailed illustrations of the bulbs’ tips and endogynes of *Dysdera erythrina* (Blackwall 1864, pl. XXVIII, Fig. 266 f; Schult 1983, 73; Roberts 1985, 61; Roberts 1998, 98; Uhl 2000, 164, Fig. 1; Kovblyuk et al. 2008, 289, Figs. 22–25; Řezáč et al. 2018) and *Dysdera crocata* (Grasshoff 1959; Roberts 1985, 61; Roberts 1998, 97; Paquin and Dupérré 2003, 71 Řezáč et al. 2007/2008, 435, Figs. 2–4; Kovblyuk et al. 2008, 289, Figs. 1–4, 8–10; Cassar and Řezáč 2021, 83 Fig. 3a) are rare, and a discussion of possible correspondences between these structures is completely lacking. To avoid misunderstandings, we would like to emphasise that we are not talking about the bulb as a whole here, but about the tip of the bulb. The publications by Arnedo & Ribera (1997, 1999) and Arnedo et al. (2000, 2007, 2009) contain relatively accurate drawings of the bulb and the endogyne as well as REM photos of the bulb tips of various *Dysdera* species. However, a possible complementarity between the bulb’s tip and the structures of the endogyne is not examined here either. Macías-Hernández et al. (2010) have shown how DNA sequence data, together with morphometric, distributional, and ecological information assists in identifying and diagnosing previously overlooked lineages. They present SEM photos of the tips of the bulbs and very precise drawings of the endogynes of different *Dysdera* species, but they don’t treat a possible complementarity between these structures. The same is true of Crespo et al. (2021), who have described eight new *Dysdera* species from the Madeira archipelago based on the integration of morphological and molecular data.

Uhl (2000, 164) provides a semi-diagrammatic drawing of a longitudinal section of the female genitalia of *Dysdera erythrina* that is doubtless absolutely correct. However, it does not demonstrate the meaning of this structure in its functional context, as Uhl’s figures do not sufficiently illustrate aspects and parts that are decisive for successful sperm transmission. With the section plane as chosen by Uhl neither the exact correspondence between the anterior diverticulum and the distal semi-circular sclerite of the male bulb can be seen, nor is the anterior median ventral passage leading from the bursa into the heavily sclerotised bilobed spermatheca recognisable. Consequently, the functional aspect of the female genitalia of *Dysdera erythrina* has been widely neglected.

In one of his numerous studies on genital mechanics and copulatory mechanisms in pholcids (Huber 1993, 1994a, b, 1995a, b, 1997, 1998, 1999, 2002; Huber & Eberhard 1997) Huber has already proposed the idea that the female might perceive the mechanical fit of those structures of the male copulatory organ that have contact (Huber 1993). Moreover, Huber (2002) pointed out that in pholcids “the procursus is innervated and may provide information for the male about its position in relation to the female.” Regrettably, Huber’s line of thought, which has a lot in common with a biosemiotic point of view (Schult et al. 2021), has not been seriously pursued by others. As Eberhard & Huber (2010, 255) state, “most studies of the functional anatomy of [spider] genitalia are unfortunately extremely typological”. Only recently does this situation seem to have improved, as is clear from the very detailed analysis of the structures of the endogyne and the function of the procursus and embolus in the pholcid species *Gertschiola neuquena* by Izquierdo et al. (2023).

The sclerites of the genital bulb are considered to be fused. As a basis for phylogenetic interpretations, Kraus (1984a) presents a detailed account of the morphological features of the different forms of male palpal organs with special consideration of those structural elements that are complementary to the epigynal area. Agnarsson et al. (2007) have reviewed the morphology of the male palpal organ in the spider family *Theridiidae* by applying a topological method that identifies homologous sclerites using their relative position. The bulb is a very complex system within which the cooperation between sclerites and inflatable areas (*haematodochae*) is very important (Quade et al. 2019). Also, the complicated torsion of the bulbs before copulation is not understandable without a description of their morphological characteristics.

Usually, the bulb has not been considered in context and as part of a specific biological sign interpretation system. This is in stark contrast to statements such as Laubichler’s (1999, 417), stressing: “What is perhaps the most striking feature of biological processes in general is their functionality, or their ‘meaningfulness’”. Huber (2004) points out the pivotal role of studies by Eberhard and West-Eberhard (1983, 1984, 1985) in demonstrating that copulatory organs are also courtship organs and “competitive signalling devices” (West-Eberhard 1984).

Concerning the evolution of the complex male and female genital structures in spiders, biosemiotics might add a new aspect to Eberhard and Huber’s model of rapid evolutionary divergence due to sexual selection by cryptic female choice. Eberhard & Huber (2010, 263–264) postulate: “Seen from the evolutionary perspective of females, the mechanical problems experienced by males that lack sense organs in their genitalia could lead to selection on females to discriminate against those males least able to achieve effective genitalic alignment, either through the stimuli received or via changes in morphology that bias male abilities to fit mechanically. The female could gain via the production of sons with superior genitalic designs. […] The female would thus be exercising sexual selection by cryptic female choice with respect to the male’s ability to adjust mechanically to her complex genitalic morphology.”

With the recent findings of innervation in the bulbs of several spider species (see above), Eberhard and Huber’s model seems all the more plausible. Seen from a biosemiotic perspective, the male’s ability to achieve effective genitalic alignment or to adjust mechanically to the female’s genitalic morphology depends on the male’s general ability to adequately interpret signs and to perform semiotic processes, i.e., it depends on its cognitive ability, which is defined as an “individual’s overall ability to acquire, retain, process, and use information” (Niemelä et al. 2013). Thus, the sexual selection by cryptic female choice as postulated by Eberhard and Huber might involve a selection of the male’s cognitive ability in general. Experimental studies on the effect of mate choice and sexual selection on the evolution of cognition in invertebrates are very rare and have produced equivocal evidence (Hollis and Kawecki 2014; Baur et al. 2019; Maggu et al. 2022). In spiders, the mate choice hypothesis for the evolution of cognition (Simons & Tibbetts 2019) has not yet been tested.

It is quite clear that a successful sperm transfer requires the correct position of the embolus and the exit opening of its ejaculatory duct. Furthermore, for the sperm transfer to be initiated there must be some signal that the correct position of the embolus has been achieved. The mechanical fitting together of the embolus and a corresponding female genital structure is the best candidate for providing such a sign. In *Argiope bruennichi*, judging by the shape of the sperm transferring part (e1 in Fig. 4D in Uhl et al. 2007) of the embolus, this seems to be the case when this part of the embolus lies completely against the inner wall of the atrium, an aspect that until now hasn’t received the attention it deserves.

The copulation behaviour of *Dysdera* has already been sufficiently described by previous authors (Cooke 1965; Helversen 1976). The insertion of the bulbs is simultaneous; the male pushes the tip of the left bulb into the right half and the tip of the right bulb into the left half of the female *bursa copulatrix*. However, it has been overlooked by other authors that in *Dysdera erythrina* one embolus intrudes a little deeper than the other and that shortly thereafter the bulbs change their position, i.e. the deeper inserted embolus is retracted while the other embolus is slightly pushed forward (unpublished observation by J. Schult on May 14, 1984). This behaviour could be explained if we assume that the morphological conditions allow only one distal sclerite of the bulb to be inserted into the anterior diverticulum at a time.

## Conclusion

The morphological conditions shown in Figs. 1, 2, 3 and 4 indicate that in *Dysdera erythrina* and *Dysdera crocata*, the bulb’s distal sclerite is anchored in the anterior diverticulum during copulation. It became clear that the distal sclerite of the bulb of *Dysdera erythrina* would not fit into the anterior diverticulum of *Dysdera crocata*, and vice versa. The hypothesis of an adaptation between these special parts of the bulb and the endogyne has been verified by microscopic examination and using SEM photographs. In the framework of biosemiotics, the bulb’s distal sclerite and, perfectly fitting to it in shape and size, the anterior diverticulum are considered morphological zones of an intraspecific sign-interpreting process that is one of the necessary prerequisites for sperm transfer. Hence these morphological elements deserve particular attention, as they are of higher taxonomic value than those morphological traits of the bulb for which a relevant role in mating and fertilisation has not been proven. In our view, Quade et al. (2019) oversimplify when they claim that “the shape of all bulb components is species specific “ because “the bulb of a male spider fits only into the genital opening of a female of the same species.“ Instead, a focus on semiotically significant characteristics, such as the complementary parts of the endogyne and the distal structures of the bulbi, would improve the merely comparative approach to morphological species classification. We want our paper to be viewed as a suggestion for further investigation in this field.
